# Ricolinostat promotes the generation of megakaryocyte progenitors from human hematopoietic stem and progenitor cells

**DOI:** 10.1186/s13287-022-02722-5

**Published:** 2022-02-05

**Authors:** Jianan Jiang, Jinhua Qin, Jisheng Li, Xiaosong Lin, Bowen Zhang, Zeng Fan, Lijuan He, Quan Zeng, Wen Yue, Min Zheng, Xuetao Pei, Yanhua Li

**Affiliations:** 1 Stem Cell and Regenerative Medicine Lab, Institute of Health Service and Transfusion Medicine, Beijing, 100850 China; 2 Experimental Hematology and Biochemistry Lab, Beijing Institute of Radiation Medicine, Beijing, 100850 China; 3 South China Research Center for Stem Cell and Regenerative Medicine, SCIB, Guangzhou, 510005 China; 4 College of Chemistry and Environmental Science, Hebei University, Baoding, 071002 Hebei China; 5 School of Medicine, University of Electronic Science and Technology of China, Chengdu, 610072 Sichuan China

**Keywords:** Ricolinostat, Hematopoietic stem and progenitor cell, Megakaryocyte progenitor, Megakaryocyte

## Abstract

**Background:**

Ex vivo production of induced megakaryocytes (MKs) and platelets from stem cells is an alternative approach for supplying transfusible platelets. However, it is difficult to generate large numbers of MKs and platelets from hematopoietic stem cells and progenitor cells (HSPCs).

**Methods:**

To optimize the differentiation efficiency of megakaryocytic cells from HSPCs, we first employed a platelet factor 4 (PF4)-promoter reporter and high-throughput screening strategy to screen for small molecules. We also investigated the effects and possible mechanisms of candidate small molecules on megakaryocytic differentiation of human HSPCs.

**Results:**

The small molecule Ricolinostat remarkably promoted the expression of PF4-promoter reporter in the megakaryocytic cell line. Notably, Ricolinostat significantly enhanced the cell fate commitment of MK progenitors (MkPs) from cord blood HSPCs and promoted the proliferation of MkPs based on cell surface marker detection, colony-forming unit-MK assay, and quantitative real-time PCR analyses. MkPs generated from Ricolinostat-induced HSPCs differentiated into mature MKs and platelets. Mechanistically, we found that Ricolinostat enhanced MkP fate mainly by inhibiting the secretion of IL-8 and decreasing the expression of the IL-8 receptor CXCR2.

**Conclusion:**

The addition of Ricolinostat to the culture medium promoted MkP differentiation from HSPCs and enhanced the proliferation of MkPs mainly by suppressing the IL-8/CXCR2 pathway. Our results can help the development of manufacturing protocols for the efficient generation of MKs and platelets from stem cells in vitro.

**Supplementary Information:**

The online version contains supplementary material available at 10.1186/s13287-022-02722-5.

## Introduction

Platelet transfusion is a critical prophylaxis or therapeutic measure for patients with hemorrhage tendency [1–3]. However, the application of platelet transfusion is severely restricted by inadequate volunteer donation, the short shelf life of platelets, and risks associated with bacterial contamination and disease transmission [4, 5]. Currently, the shortage of platelets and increased demand for platelets are growing. Ex vivo manufacture of induced platelets from stem cells is being developed as an alternative approach for supplying transfusible platelets [6–9]. Additionally, infusion of induced megakaryocytes (MKs) or their progenitor cells derived from stem cells may improve platelet counts in the peripheral blood of patients with severe thrombocytopenia [10–12]. Thus, methods are needed for the in vitro efficient manufacture of stem cell-derived MKs and platelets.

Several types of stem cells, including human pluripotent stem cells and cord blood (CB)-derived hematopoietic stem and progenitor cells (HSPCs), can differentiate into MKs and platelets [9, 13–19]. Clinically applicable MKs and platelets from human pluripotent stem cells without gene modification are currently being evaluated [16]. Recently, expandable MK cell lines induced by overexpression of several genes in human pluripotent stem cells have been manufactured and exhibited the capacity for scalable platelet generation [9, 20]. Compared with using human pluripotent stem cells as seed cells, which have a teratoma risk, induced MKs and platelets derived from CB HSPCs are more acceptable for patients with bleeding risk in the clinic. However, the limited numbers and multiple blood lineage differentiation potential of HSPCs in CB samples have impeded the large-scale generation of MKs and platelets. Accumulating evidence suggests that aging or expansion of HSPCs can be regulated by small molecules or extracts of traditional Chinese herbs [21–23], which is crucial for generating large numbers of megakaryocytic cells. Another strategy for efficiently producing MKs and platelets from CB HSPCs is to improve megakaryocytic fate commitment and amplify MK progenitors (MkPs) at an early differentiation stage.

Preliminary screening of effective agents that can regulate MK-specific gene expression in megakaryocytic cell lines will aid in the discovery of chemicals to enhance the MK fate from stem cells. The platelet factor 4 (PF4) gene is expressed exclusively in megakaryocytic cells, proplatelets, and platelets [24, 25]. Accumulating evidence has shown that the PF4 gene is a critical marker of megakaryocytic differentiation, which is regulated by several MK-specific transcriptional factors. Hence, we first screened small molecules that could promote the expression of PF4 by employing a PF4-promoter reporter and high-throughput screening (HTS) strategy. We found that a specific small molecule, Ricolinostat, enhanced PF4 gene expression and efficiently promoted the generation of MkPs mainly by enhancing MkP fate commitment from HSPCs and promoting MkP proliferation. These MkPs generated from Ricolinostat-induced HSPCs differentiated into mature MKs and platelets. Mechanistically, we found that Ricolinostat enhances MkP fate mainly by inhibiting the expression and interaction of IL-8 and its receptor, which suppress megakaryopoiesis [26, 27].

## Materials and methods

### Small molecule screening

The human PF4 promoter-GFP reporter vector, PLVX-PF4-promoter-GFP, was constructed as previously reported [28]. The megakaryocytic cell lines MEG01 and K562 were transfected with PLVX-PF4-promoter-GFP and selected with puromycin. K562 cells with PLVX-PF4-promoter-GFP were resuspended in culture medium (2 × 10^4^ cells/mL) and aliquoted into 96-well plates (PerkinElmer, Waltham, MA, USA). The compounds were added immediately after plating. The cells were cultured at 37 °C in 5% CO_2_ for 48 h and analyzed using a Cell Insight CX5 HCS platform (Thermo Fisher Scientific, Waltham, MA, USA) [29]. An epigenetic compound library composed of 120 chemical compounds was purchased from Selleckchem (Houston, TX, USA).

### Purification of human CB-derived CD34^+^ cells

Human CB samples were obtained from the umbilical cord after delivery of normal pregnancies with informed consent from the patient. The study was approved by the ethics committee. We first separated low-density mononuclear cells using Ficoll-Hypaque density gradient centrifugation (1.077 g/L; TBD Science, Tianjin, China), and then purified CB CD34^+^ cells from mononuclear cells by positive selection using a MACS CD34 MicroBead Kit (Miltenyi Biotec, Gladbach Bergisch, Germany) according to the manufacturer’s instructions [30, 31]. More than 90% of enriched cells were CD34-positive, as confirmed by fluorescence-activated cell sorting (FACS).

### Differentiation of MKs from HSPCs

Based on previous studies [4], a stepwise three-stage protocol was used to induce HSPC differentiation in MKs. In stage I, isolated HSPCs were expanded in StemSpan™ SFEM medium (STEMCELL Technologies, Vancouver, Canada) containing recombinant human stem cell factor (SCF, 50 ng/ml, Peprotech, Rocky Hill, NJ, USA), recombinant human thrombocytopoietin (TPO, 50 ng/mL, Peprotech), interleukin (IL)-3 (20 ng/mL, Peprotech), and recombinant human Flt3-Ligand (FLT3-L, 50 ng/mL, Peprotech). In stage II, the MK specification stage, after expansion for 7 days, the cells were cultured in StemSpan™ SFEM medium containing SCF (50 ng/mL), TPO (50 ng/mL), IL-3 (20 ng/mL), IL-6 (50 ng/mL, Peprotech), and IL-11 (20 ng/mL, Peprotech), with or without 2 μM Ricolinostat. In stage III, as the MK maturation stage, after 7-day cell specification, cells were washed and cultured in StemSpan™ SFEM medium containing SCF (10 ng/mL), TPO (50 ng/mL), IL-3 (20 ng/mL), IL-6 (20 ng/mL), and IL-11 (20 ng/mL). The medium was refreshed every 2–3 days.

### Colony-forming unit-MK assay

To detect and quantify human MK progenitors, on day 7 of stage II, 40,000 cells were plated in a serum-free collagen-based system using the MegaCult™-C Complete Kit with Cytokines (STEMCELL Technologies) according to the manufacturer’s instructions [32]. After incubation for 12–14 days on double-chamber culture slides, megakaryocytic colonies were stained using the antibody provided with the kit. Colony-forming unit (CFU)-MKs were cultured and stained according to the instructions of the MegaCult™-C Complete Kit with Cytokines. Megakaryocytes and platelets both express glycoprotein llb/llla (CD41) and become pink after fixation and staining. Evans blue counterstaining was used to stain the cell nuclei as light blue. Therefore, CFU-MK appears as a group of cells with pink cell membranes and blue cell nuclei. The number of cells contained in different CFU-MK colonies ranged from three to several hundred. For convenience, these colonies were divided into three categories based on their sizes: small (3–20 cells in each colony), medium (21–49 cells in each colony), or large (≥ 50 cells in each colony).

### Flow cytometry analysis and cell sorting

Single-cell suspensions were stained with cell surface antigens in phosphate-buffered saline (PBS) at 4 °C for 40 min. The stained cells were analyzed or sorted using BD FACSCalibur™ (BD Biosciences, Franklin Lakes, NJ, USA), and the data were analyzed using FlowJo software (TreeStar, Ashland, OR, USA) [16]. The sources of antibodies are listed in Additional file 1: Table S1.

### RT-PCR and quantitative real-time PCR analyses

Total RNA was isolated using an RNeasy extraction kit (Qiagen, Hilden, Germany). RNA was reverse-transcribed using Superscript II reverse transcriptase (Invitrogen, Carlsbad, CA, USA), according to the manufacturer’s instructions. Reverse transcription (RT)-PCR was performed using Taq polymerase (TaKaRa, Shiga, Japan). Quantitative RT-PCR (qRT-PCR) was performed using SYBR Green real-time PCR master mix (TOYOBO, Osaka, Japan) on a Bio-Rad iQ5 Real-Time PCR detection system (Hercules, CA, USA). Data were analyzed using the ΔΔCt method [33, 34]. The primers are shown in Additional file 1: Table S2.

### Statistics

All statistical data are expressed as the mean ± standard error of the mean (SEM). Statistical analyses were performed using GraphPad Prism version 7 software (GraphPad, Inc., La Jolla, CA, USA). For most statistical evaluations, a two-tailed Student’s *t*-test was used to calculate the statistical probability. Statistical significance was set at *p* < 0.05. All results were replicated in at least three independent experiments.

## Results

### Phenotypic screen identified small molecules that enhanced PF4 expression

**Fig. 1:**
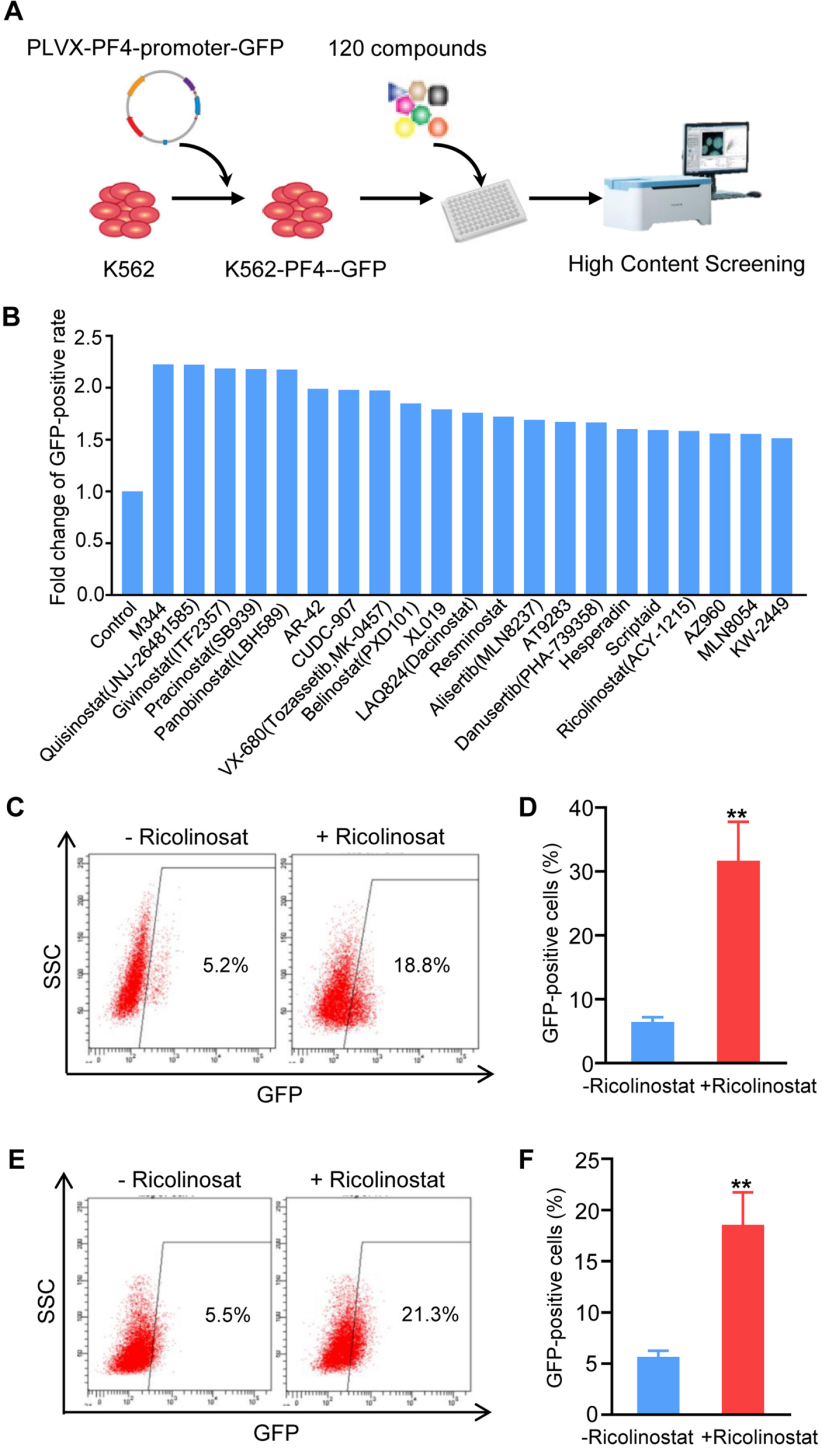
Screening of small molecules. **A** Schematic representation of the small molecule screening platform. **B** List of small molecule compounds with a fold increase in GFP-positive rate of more than 1.5 compared with the control group, which was screened by a high-content screening platform. **C**–**F** Effect of Ricolinostat on GFP expression in MEG01 and K562 cell lines. **C** Flow cytometry was used to detect the change of the GFP-positive rate in K562 cells. **D** Percentage of GFP-positive cells in K562 cells was quantified based on three independent experiments. **E** Flow cytometry was used to detect the change in the GFP-positive rate in MEG01 cells. **F** Percentage of GFP-positive cells in MEG01 cells was quantified based on three independent experiments. Results are expressed as the mean ± SEM from three independent experiments. Unpaired Student’s *t*-test, **p < 0.01

Previous studies demonstrated that PF4 is a lineage-specific marker of MK development [4, 28, 35]. Thus, we utilized PF4-GFP as a reporter system to visualize the expression level of PF4. To identify chemical compounds with the potential to enhance PF4 expression, we developed an HTS assay using a human myeloleukemia K562 cell line carrying a PF4-GFP reporter and evaluated GFP expression using a high-content screening platform after 48 h of culture with different small molecule supplementation (Fig. 1A). Using this assay, we screened a library of 120 small molecules and identified 21 candidates that increased the percentage of GFP^+^ cells by at least 1.5-fold (Fig. 1B). These 21 candidate molecules were further screened and evaluated according to the expression of GFP under the PF4-promoter in K562 cells by flow cytometry; we found that the histone deacetylase (HDAC) 6 inhibitor Ricolinostat had the strongest potential to increase the percentage of GFP^+^ cells by 2.62-fold (Additional file 1: Supplemental Fig. 1). Independent replicated experiments revealed a similar increase in the GFP^+^ cell percentages in K562 and MEG01 cell lines treated with Ricolinostat treatment (Fig. 1C–F). These data indicate that Ricolinostat can regulate PF4 expression and enhance the differentiation of MKs.

### Ricolinostat promoted megakaryocytic progenitor cell differentiation from human CB HSPCs

**Fig. 2:**
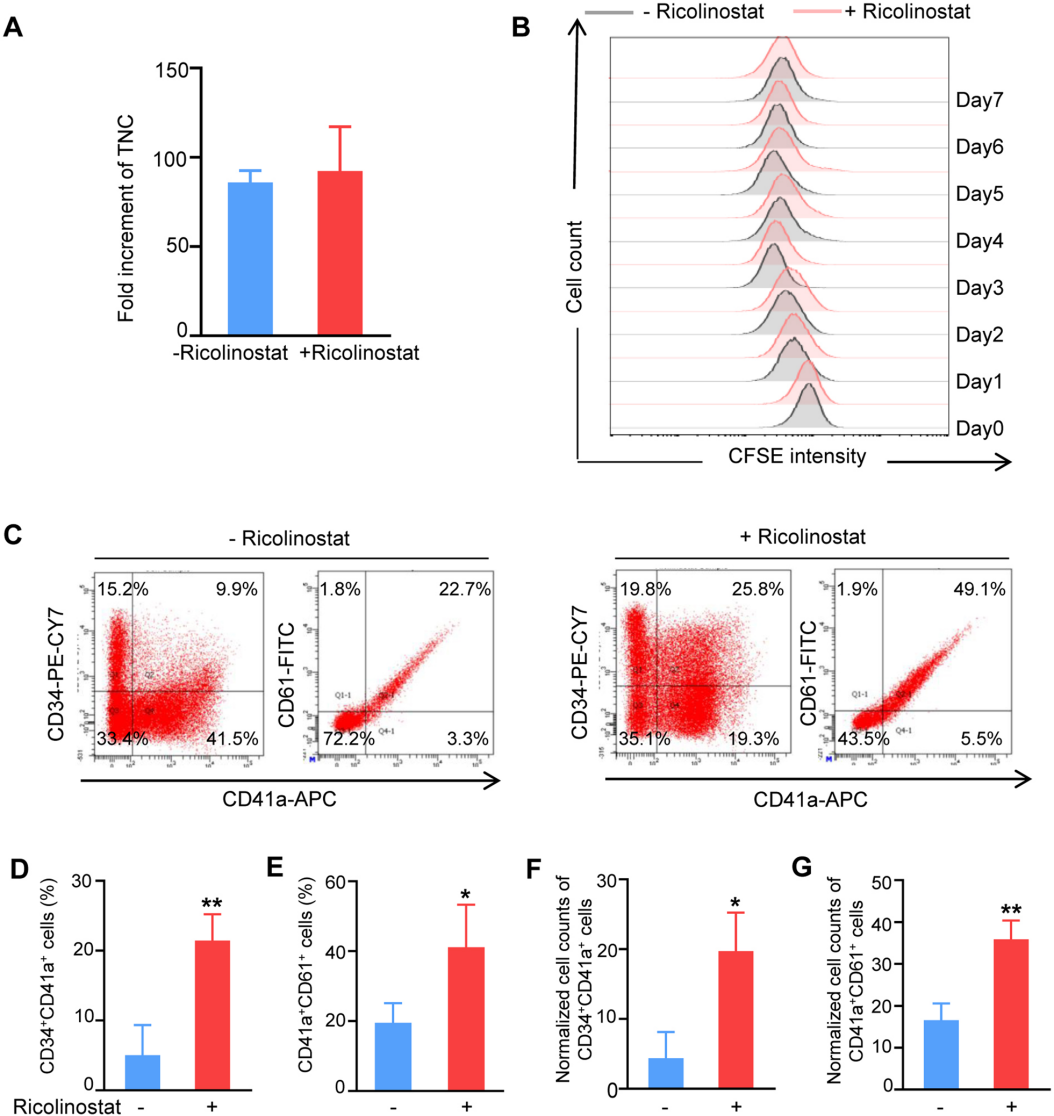
Effects of Ricolinostat on megakaryocytic differentiation of HSPCs. **A** Absolute counts of total nucleated cells at day 7 of Ricolinostat treatment were calculated and normalized to the initial HSPCs. **B** CFSE time series obtained from cells at the end of the first stage. All cells obtained from the first stage were labeled with CFSE on the first day of the second stage (day 0). CFSE fluorescence intensity was detected by flow cytometry for 7 consecutive days. **C** Flow cytometry analysis of CD34, CD41a, and CD61 expression in cells on day 7 of Ricolinostat treatment. **D** Percentage of CD34^+^CD41a^+^ cells on day 7 of Ricolinostat treatment. **E** Percentage of CD41a^+^CD61^+^ cells on day 7 of Ricolinostat treatment. **F** Absolute counts of CD34^+^CD41a^+^ cells on day 7 of Ricolinostat treatment were calculated and normalized to the initial HSPCs. **G** Absolute counts of CD41a^+^CD61^+^ cells on day 7 of Ricolinostat treatment were calculated and normalized to initial HSPCs. Results are expressed as the mean ± SEM from three independent experiments. Unpaired Student’s *t*-test, *p < 0.05; **p < 0.01

To investigate the effects of Ricolinostat on megakaryocytic differentiation of human HSPCs, enriched CB CD34^+^ cells were expanded for 7 days and then transferred into megakaryocytic differentiation medium with or without Ricolinostat for another 7 days. Ricolinostat supplementation showed no significant increase in the total nucleated cell (TNC) number in each well compared to that in the control group (Fig. 2A). To directly evaluate the proliferation rate of these differentiated cells during megakaryocytic induction, CFSE was added to the induction medium and monitored in the induced cells by flow cytometry each day. The detection results of CFSE labeling indicated that Ricolinostat did not significantly affect total nucleated cell proliferation (Fig. 2B). We then analyzed the percentage of MkPs and MKs after the 7-day induction process using flow cytometry. Notably, Ricolinostat significantly increased the percentages of CD34^+^CD41a^+^ MkPs and CD41a^+^CD61^+^ MKs by approximately 2.6- and 1.11-fold, respectively, compared to the controls (Fig. 2C–E). Consistently, the numbers of CD34^+^CD41a^+^ cells and CD41a^+^CD61^+^ cells were remarkably increased by Ricolinostat treatment (Fig. 2F–G).

**Fig. 3:**
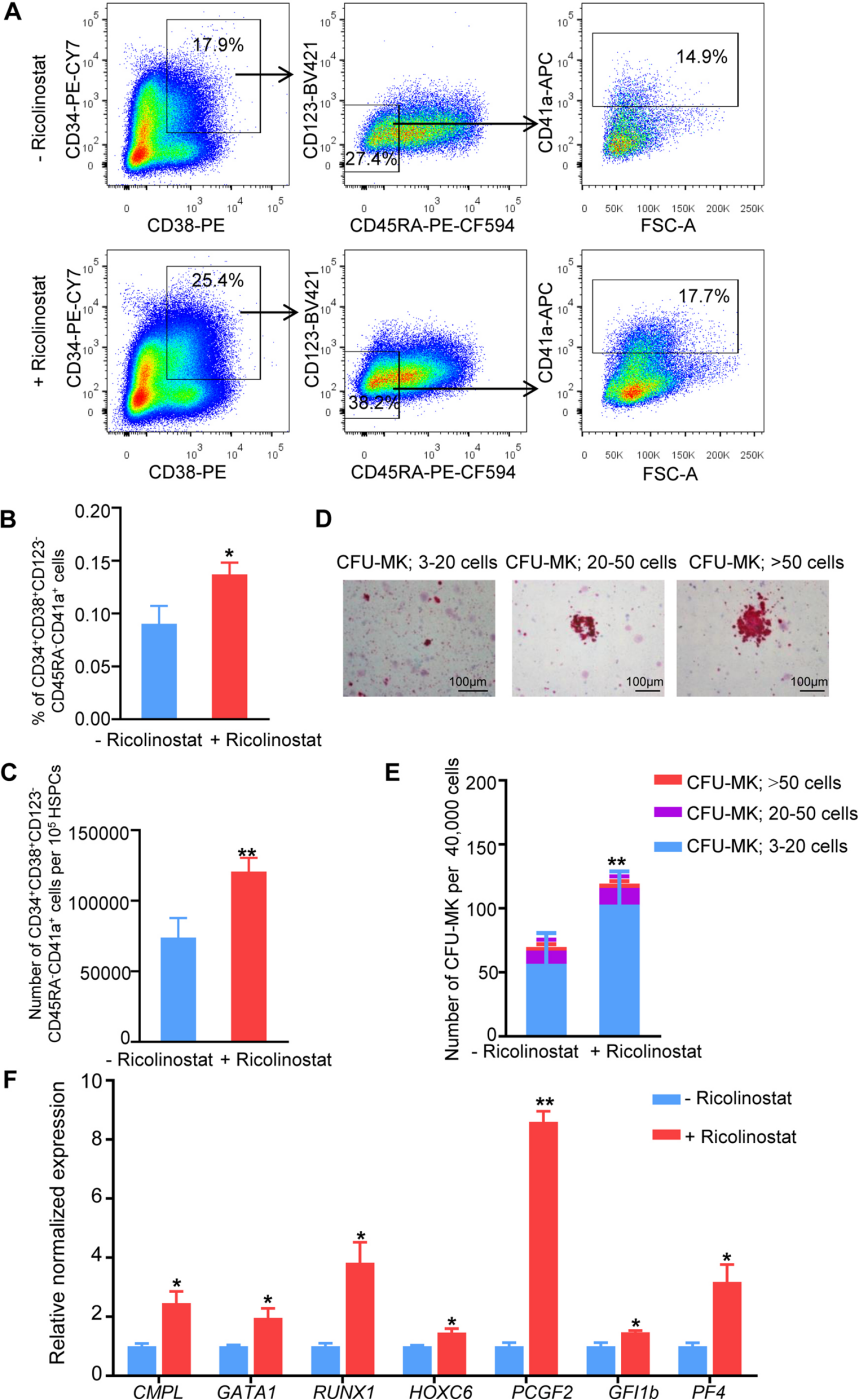
Ricolinostat promoted MkP differentiation from HSPCs. **A** Flow cytometry analysis of changes in MkP (CD34^+^CD38^+^CD45RA^−^CD123^−^CD41a^+^) cell ratios. **B** Percentage of CD34^+^CD38^+^CD45RA^−^CD123^−^CD41a^+^ cells on day 7 of Ricolinostat treatment. **C** Cell number of CD34^+^CD38^+^CD45RA^−^CD123^−^CD41a^+^ cells from initial 10^5^ HSPCs on day 7 of Ricolinostat treatment. **D** CFU-MK were identified by staining with CD41 and subclassified as follows: small (3–20 cells); medium (20–50 cells); large (≥ 50 cells). Scale bars, 100 μm. **E** Absolute number of CFU-MK in different treatment conditions. **F** Expression analysis of some MkP-related genes in Ricolinostat-treated and -untreated cells on day 7 by qRT -PCR. Results are expressed as the mean ± SEM from three independent experiments. Unpaired Student’s *t*-test, *p < 0.05; **p < 0.01

To further determine the role of Ricolinostat in inducing the generation of MkPs from CB HSPCs, we used five cell surface markers, CD34^+^, CD38^+^, CD45RA^−^, CD123^−^, and CD41a^+^, to strictly define MkPs. Ricolinostat supplementation significantly increased the percentage and number of CD34^+^CD38^+^CD45RA^−^CD123^−^CD41a^+^ MkPs (Fig. 3A–C). We then performed a CFU-MK assay to evaluate the role of Ricolinostat in the enhancing MkP production. Ricolinostat-treated groups generated more MK colonies compared to the controls (Fig. 3D–E), indicating that Ricolinostat facilitated the differentiation of CB HSPCs into MkPs. Furthermore, the qRT-PCR results revealed that the gene expression levels of key MK transcriptional factors, including GATA Binding Protein 1 (*GATA1*)*,* RUNX family transcription Factor 1 (*RUNX1*)*,* homeobox C6 (*HOXC6*), polycomb group ting finger 2 (*PCGF2*), and growth factor-independent protein 1B (*GFI1b*), were significantly upregulated by Ricolinostat (Fig. 3F). These results suggest that Ricolinostat can promote MkP differentiation in human CB HSPCs.

### Ricolinostat promoted MkP generation by enhancing differentiation and proliferation of MkPs from HSPCs

**Fig. 4:**
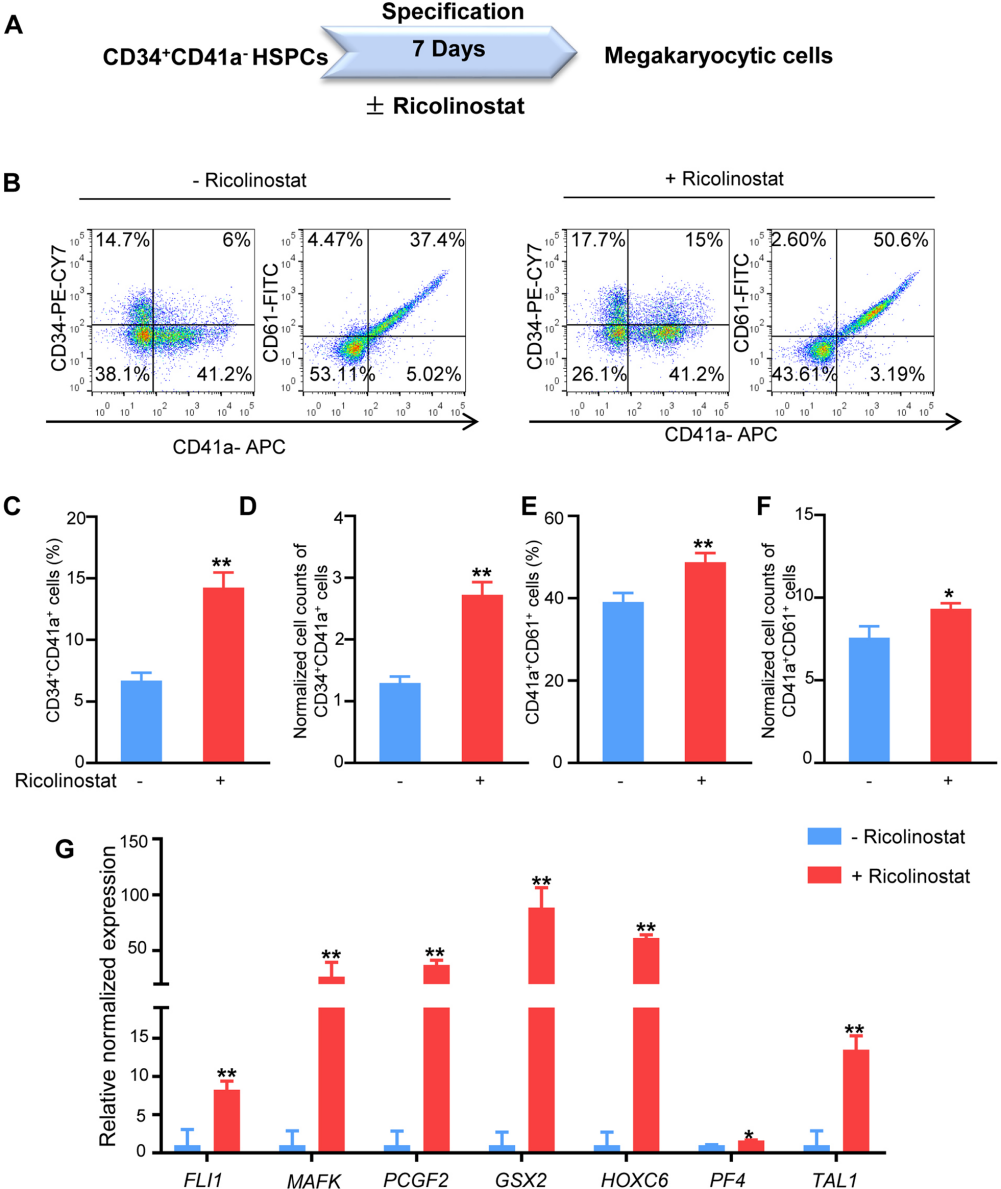
Ricolinostat promoted megakaryocytic specification of CD34^+^CD41a^−^ cells. **A** Schematic diagram illustrating that CD34^+^CD41a^−^ cells were sorted from expanded HSPCs and then treated with Ricolinostat for 7 days. **B** Flow cytometry analysis of CD34, CD41a, and CD61 expression in Ricolinostat-treated CD34^+^CD41a^−^ cells. **C** Percentage of CD34^+^CD41a^+^ cells in Ricolinostat-treated CD34^+^CD41a^−^ cells. **D** Absolute counts of CD34^+^CD41a^+^ cells were calculated and normalized to the initial sorted CD34^+^CD41a^−^ cells. **E** Percentage of CD41a^+^CD61^+^ cells in Ricolinostat-treated CD34^+^CD41a^−^ cells. **F** Absolute counts of CD41a^+^CD61^+^ cells were calculated and normalized to the initial sorted CD34^+^CD41a^−^ cells. **G** qRT-PCR analysis of some MkP-related genes in Ricolinostat-treated and -untreated CD34^+^CD41a^−^ cells. Results are expressed as the mean ± SEM from three independent experiments. Unpaired Student’s *t*-test, *p < 0.05; **p < 0.01

In vitro induction of MkP production from CB HSPCs may involve two biological events: differentiation of HSPCs toward MkPs and expansion of MkPs. To dissect the role of Ricolinostat in the MkP generation process, we sorted CD34^+^CD41a^−^ cells and CD34^+^CD41a^+^ cells by FACS and cultured the cells with or without Ricolinostat for 7 days (Fig. 4A). Ricolinostat supplementation in the MK culture medium significantly increased the percentage and number of CD34^+^CD41a^+^ cells and CD41a^+^CD61^+^ cells derived from CD34^+^CD41a^−^ HSPCs (Fig. 4B–F), indicating that more megakaryocytic cells were induced and differentiated from CD34^+^CD41a^−^ HSPCs. Additionally, the qRT-PCR results revealed that MK-related genes, including GS homeobox 2 (*GSX2*), *PCGF2*, *HOXC6*, and T-cell acute lymphocytic leukemia 1(*TAL1)* were significantly increased in the Ricolinostat-treated group (Fig. 4G), supporting that Ricolinostat enhanced megakaryocytic fate commitment from CD34^+^CD41a^−^ HSPCs.

**Fig. 5:**
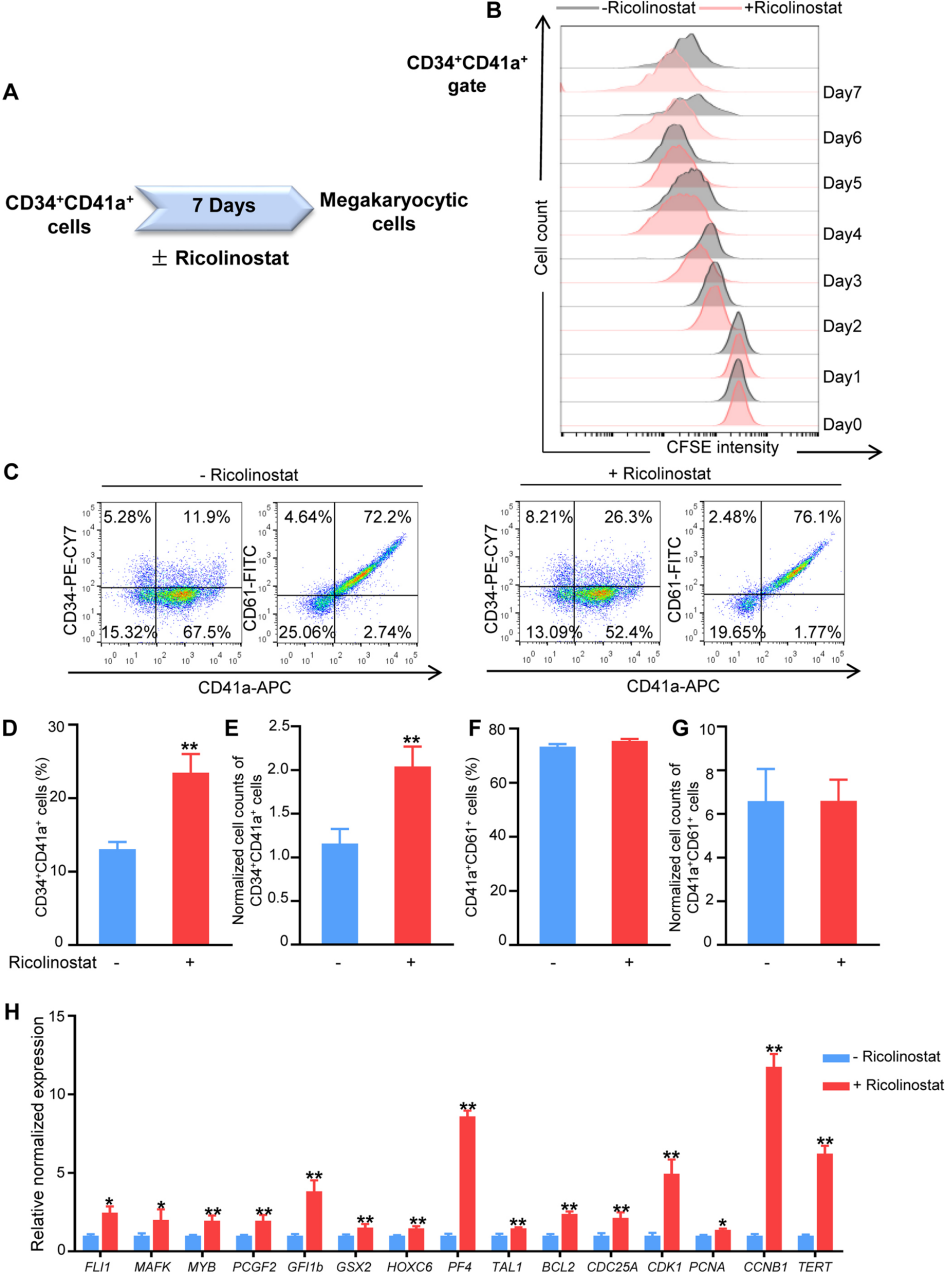
Ricolinostat enhanced proliferation of CD34^+^CD41a^+^ cells. **A** Schematic diagram illustrating CD34^+^CD41a^+^ cells sorted from expanded HSPCs and then treated with Ricolinostat for 7 days. **B** CFSE time series obtained from CD34^+^CD41a^+^ populations. All cells obtained from the first stage were labeled with CFSE on the first day of the second stage (day 0). The CFSE fluorescence intensity of CD34^+^CD41a^+^ cells was detected by flow cytometry for 7 consecutive days. **C** Flow cytometry analysis of CD34, CD41a, and CD61 expression in Ricolinostat-treated CD34^+^CD41a^+^ cells. **D** Percentage of CD34^+^CD41a^+^ cells in Ricolinostat-treated CD34^+^CD41a^+^ cells. **E** Absolute counts of CD34^+^CD41a^+^ cells were calculated and normalized to the initial sorted CD34^+^CD41a^+^ cells. **F** Percentage of CD41a^+^CD61^+^ cells in Ricolinostat-treated CD34^+^CD41a^+^ cells. **G** Absolute counts of CD41a^+^CD61^+^ cells were calculated and normalized to the initial sorted CD34^+^CD41a^+^ cells. **H** qRT-PCR analysis of some MkP-related genes in Ricolinostat-treated and -untreated CD34^+^CD41a^+^ cells. Results are expressed as the mean ± SEM from three independent experiments. Unpaired Student’s *t*-test, *p < 0.05; **p < 0.01

We then investigated the role of Ricolinostat in CD34^+^CD41a^+^ MkP expansion (Fig. 5A). The CFSE labeling assay was performed to monitor the proliferation of CD34^+^CD41a^+^ cells each day. Notably, CD34^+^CD41a^+^ cells treated with Ricolinostat displayed decreased CFSE intensity from day 3, particularly on days 6 and 7 (Fig. 5B), indicating that Ricolinostat enhanced the cell cycle speed of MkPs. Correspondingly, Ricolinostat addition to the medium led to a significant increase in the percentage and number of CD34^+^CD41a^+^ cells by approximately 79% and 76%, respectively (Fig. 5C–E), calculated as the percentage or number of CD34^+^CD41a^+^ cells in the Ricolinostat-treated group minus that of the non-treated group, and then divided by the percentage or number of CD34^+^CD41a^+^ cells in the non-treated group. Ricolinostat did not significantly affect the percentage and number of CD41a^+^CD61^+^ cells derived from CD34^+^CD41a^+^cells (Fig. 5F–G). The qRT-PCR results showed that the expression levels of MK-related genes such as *HOXC6*, *PCGF2*, *GSX2*, and Fli-1 proto-oncogene (*FLI1)* were significantly enhanced by Ricolinostat (Fig. 5H). Ricolinostat treatment remarkably increased the expression of cell proliferation-related genes, such as B-cell lymphoma-2 (*BCL2*), cyclin-dependent kinase 1 (*CDK1*), *CYCLIN B1* (CCNB1), and telomerase reverse transcriptase (*TERT*) (Fig. 5H). These results indicate that Ricolinostat promotes MkP fate commitment mainly by enhancing the differentiation and expansion of MkPs from CB HSPCs.

### Ricolinostat enhanced MkP fate by inhibiting expression of IL-8 and its receptor CXCR2

**Fig. 6:**
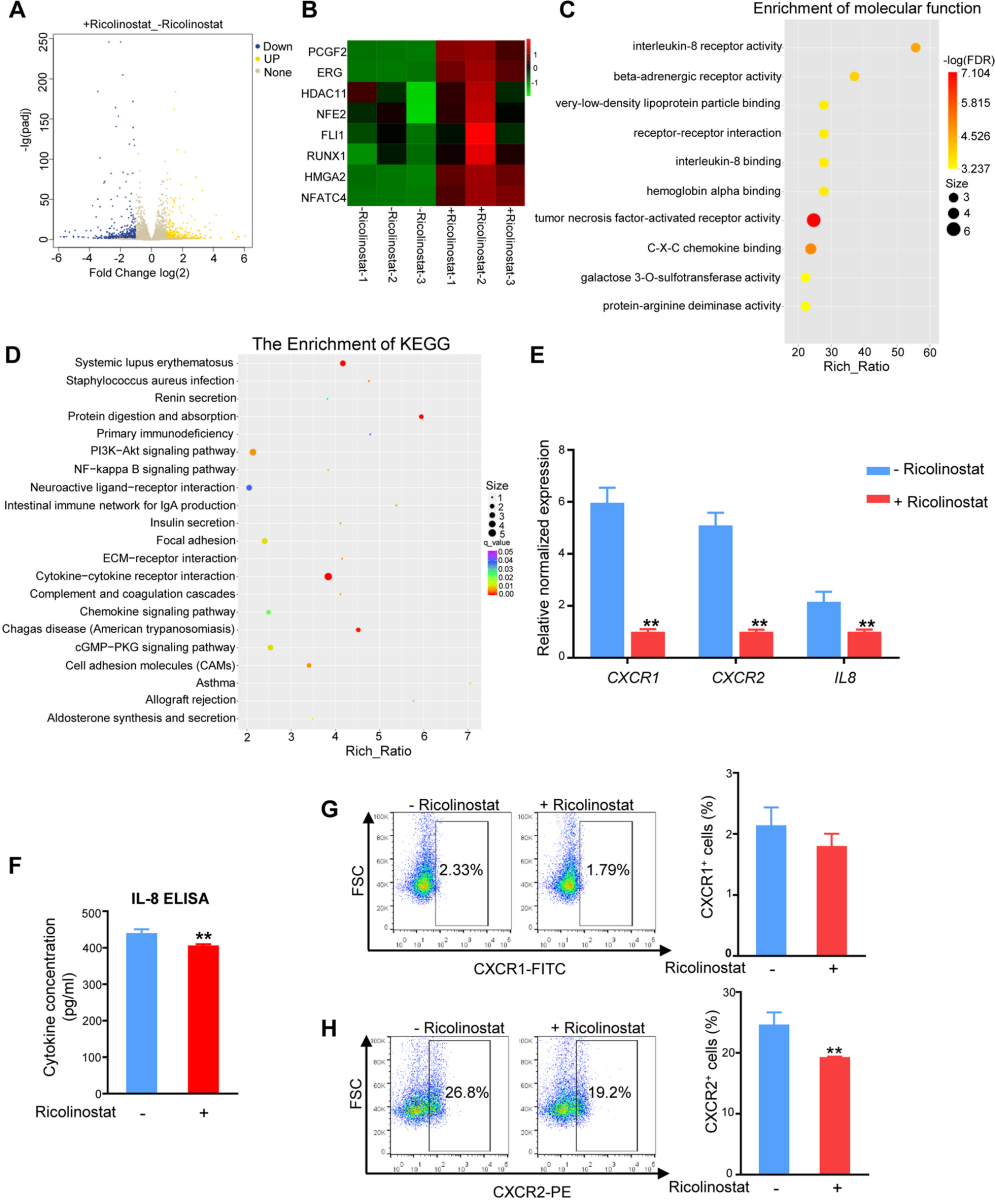
Ricolinostat might promote MkP differentiation by inhibiting IL-8 and its receptors. **A** Volcano map of differentially expressed genes. The abscissa is the fold change in gene expression in different experimental groups/different samples, and the ordinate is the significant degree of expression change. Blue represents significantly downregulated genes, and yellow represents significantly upregulated genes. Gray represents genes that were not significantly different. **B** Heat map of RNA-seq data for the indicated MK-related genes. **C** Gene Ontology analysis of downregulated genes in Ricolinostat-treated group compared to the untreated group. **D** KEGG analysis of downregulated genes in Ricolinostat-treated group compared to the untreated group. **E** qRT-PCR analysis of *CXCR1*, *CXCR*2, and *IL-8* expression. **F** Expression of IL-8 in the culture supernatant on day 7 of Ricolinostat treatment measured by ELISA. **G** Flow cytometry analysis of CXCR1 expression on day 7 of Ricolinostat treatment. **H** Flow cytometry analysis of CXCR2 expression on day 7 of Ricolinostat treatment. Results are expressed as the mean ± SEM from three independent experiments. Unpaired Student’s *t*-test, **p < 0.01

To explore the downstream mechanism of Ricolinostat in the enhancing MkP fate, we performed RNA-seq analysis. The addition of Ricolinostat to the medium led to 726 differentially expressed genes (DEGs) compared to the controls (Fig. 6A). Notably, MK-related genes, such as *PCGF2*, ETS-related gene (*ERG*), and high-mobility group AT-hook 2 (*HMGA2*) were significantly upregulated by Ricolinostat (Fig. 6B). Additionally, 56.2% of the DEGs were downregulated by Ricolinostat treatment, which was predominantly involved in molecular functions, including IL-8 receptor activity, receptor–receptor interaction, and IL-8 binding (Fig. 6C). Kyoto Encyclopedia of Genes and Genomes (KEGG) analysis showed that genes downregulated in Ricolinostat-treated cells were enriched in pathways such as “cytokine–cytokine receptor interaction,” “chemokine signaling pathway,” and “PI3K − Akt signaling pathway” (Fig. 6D).

It has been reported that IL-8 and its receptors C-X-C motif chemokine receptor 1 (CXCR1) and C-X-C motif chemokine receptor 2 (CXCR2) act as negative regulators of MKs [26, 36]. Therefore, we aimed to verify the role of Ricolinostat in regulating IL-8 and its receptors. The qRT-PCR results showed that the gene expression levels of IL-8 and its receptors CXCR1 and CXCR2 were significantly suppressed by Ricolinostat (Fig. 6E). We next performed ELISA to quantify the IL-8 level in the cell culture supernatants and found that the amount of secreted IL-8 was decreased in the Ricolinostat-treated group (Fig. 6F). Moreover, flow cytometry analysis showed that CXCR2-positive cells were decreased after Ricolinostat treatment (Fig. 6G–H). These results indicate that Ricolinostat suppresses the interaction of IL-8 and its receptor CXCR2 by inhibiting the expression of this ligand and receptor, thereby decreasing its negative regulatory role on MKs and enhancing MkP fate commitment.

### MkPs generated from Ricolinostat-treated HSPC gave rise to mature MKs

**Fig. 7:**
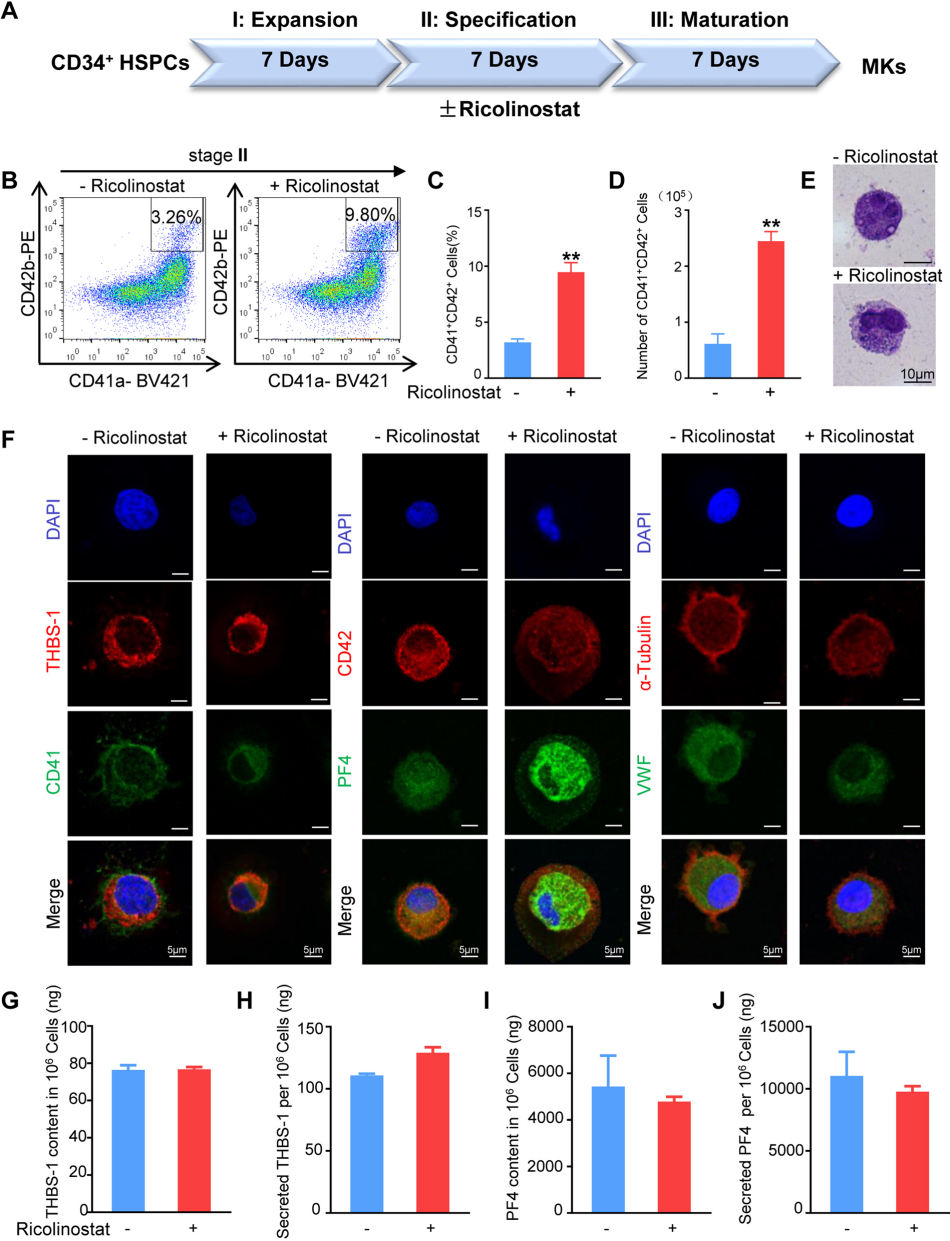
Induction of MkPs into MKs. **A** Schematic representation of the stepwise protocol for HSPC differentiation into MKs. CD34^+^ HSPCs were cultured for 7 days in the presence of SCF, TPO, IL-3, IL-6, and FLT3-L for HSPC expansion (stage I). Cells obtained from the first stage were cultured for 7 days in the presence of SCF, TPO, IL-3, IL-6, and IL-11, with or without Ricolinostat for megakaryocytic lineage specification (stage II). Megakaryocytic lineage cells obtained from the second stage were cultured without Ricolinostat for 7–14 days in the presence of cytokines for MK maturation and platelet production (stage III). **B** Flow cytometry analysis of CD41a and CD42b expression in cells obtained from the third stage on day 21. **C** Percentage of CD41a^+^CD42b^+^cells on day 21. **D** Number of CD41a^+^CD42b^+^cells from per 10^5^ HSPCs on day 21. **E** Identification of morphology and structure of MKs by Wright's Giemsa staining. Scale bars, 10 μm. **F** Immunofluorescence analysis showed the expression and location of MK-specific markers CD41, CD42b, α-Tubulin, vWF, PF4, and THBS-1 in cells on day 21. Scale bars, 5 μm. **G**, **H** Expression of THBS-1 in the culture supernatant and cells on day 21 measured by ELISA. **I**, **J** Expression of PF4 in the culture supernatant and cells on day 21 measured by ELISA. Results are expressed as the mean ± SEM from three independent experiments. Unpaired Student’s *t*-test, *p < 0.05

It was previously reported that HDAC6 inhibition leads to defects in MK maturation [32]. Thus, we withdrew Ricolinostat from the MK differentiation medium to observe whether these differentiated cells could further differentiate into mature MKs and release platelets after stage II induction (Fig. 7A). The percentage and number of CD41a^+^CD42b^+^ cells produced by Ricolinostat treatment in stage II increased approximately 1.96- and 3.14-fold compared to the group without Ricolinostat addition (Fig. 7B–D). Large polyploid MKs were detected by Giemsa staining in these two groups (Fig. 7E). Ultrastructural analysis by electron microscopy showed that the differentiated cells from both groups had typically lobulated nuclei, developing a demarcation membrane system (cytoplasmic cell membrane supply for platelet release), cytoplasmic multi-vesicular bodies (precursors to platelet granules), and mature granules (Additional file 1: Supplemental Fig. 2A). We analyzed the nuclear content of MKs on day 21 by flow cytometry and found that Ricolinostat treatment had little effect on MK ploidization (Additional file 1: Supplemental Fig. 2B–C). Differentiated MKs from the two groups showed characteristic granular accumulation of THBS-1, PF4, von Willebrand factor (vWF) in the cytoplasm, and CD41 and CD42b staining on the membrane (Fig. 7F). Additionally, we performed ELISA to quantify the synthesis and secretion of THBS-1 and PF4 in 1 × 10^6^ MKs. The amounts of intracellular and secreted THBS-1 and PF4 were similar between the two groups (Fig. 7G–J). These results indicate that Ricolinostat supplementation in stage II did not suppress MK maturation.

**Fig. 8:**
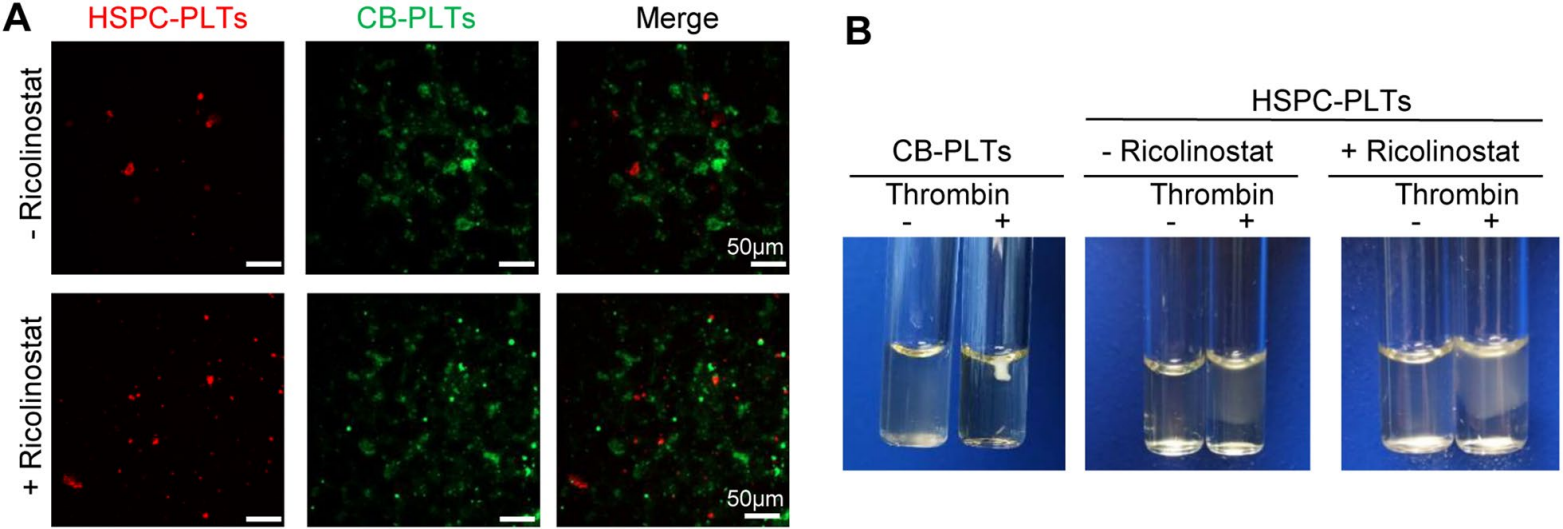
Functional verification of platelets. **A** Platelet aggregation status in different treatment groups. Scale bars, 50 μm. **B** Clot formation and retraction. Platelet-depleted human plasma was added to platelets derived from different treatments or platelets derived from cord blood. Thrombin was then added to the suspensions to induce clot formation and retraction

Furthermore, our data showed that when Ricolinostat was withdrawn in stage III, the proplatelet formation ability of MKs was not altered by Ricolinostat treatment in stage II. However, if Ricolinostat remained in stage III, the proplatelet formation ability of MKs was inhibited (Additional file 1: Supplemental Fig. 3). Platelet yields were characterized using flow cytometry and immunostaining. Both groups released platelets with surface markers including CD41, CD42b, CD61, and major alpha-granule proteins, including THBS-1, PF4, and vWF (Additional file 1: Supplemental Fig. 4A–B). Moreover, platelet function assays revealed that platelets derived from Ricolinostat-treated or control groups, together with human blood platelets, formed aggregates in response to thrombin stimulation (Fig. 8A). Platelets derived from both groups could form platelet plugs and promote fibrin clot formation and retraction (Fig. 8B). Taken together, the generated megakaryocytic cells under induction conditions with Ricolinostat in stage II further efficiently differentiated into mature MKs and produced functional platelets in vitro.

## Discussion

In this study, we aimed to improve megakaryocytic fate commitment at the early differentiation stage of CB HSPCs. Previous studies suggested that PF4 serves as a lineage-specific marker for MK development [4, 28, 35]. Bioinformatic analysis from the BloodSpot database also showed that the expression of PF4 was restricted in MkPs and MKs (Additional file 1: Supplemental Fig. 5). Thus, we first employed a PF4 promoter with a GFP reporter to screen small molecules that could enhance its expression. By screening a bank of 120 small molecules that regulate epigenetic factors using HTS, we identified 21 candidate chemical compounds that promoted expression in megakaryocytic cell lines. After the second round of screening and evaluation by flow cytometry, we found that the small molecule Ricolinostat had the strongest capacity to enhance PF4 gene expression, indicating that this small molecule enhances megakaryocytic cell fate from HSPCs.

Improving the efficiency of MkP or MK precursor generation from HSPCs is a critical step in inducing functional MK and platelet production in vitro. Previous studies indicated that an MK precursor population with strong MK differentiation potential can be enriched by subculturing the cells with bone marrow mesenchymal stromal cells or StemRegenin 1 treatment [37, 38]. It remains challenging to increase the committed differentiation of MkPs from HSPCs using new inducing agents. Encouragingly, Ricolinostat enhanced MkP generation from CB HSPCs, as evidenced by the increased percentage of MkPs and improved MK colony-forming capability. A recent report indicated that several new transcriptional factors are involved in regulating MkP differentiation from HPSCs [39]. We also found that Ricolinostat remarkably upregulated the expression of some transcription factor genes involved in megakaryopoiesis, such as *PCGF2* and *HOXC6*. Increased production of MkPs from CB HSPCs by Ricolinostat may involve two biological events, including enhanced differentiation of HSPCs toward MkPs and proliferation of MkPs. To dissect the role of Ricolinostat in MkP generation, we sorted two cell populations, CD34^+^CD41a^−^ cells and CD34^+^CD41a^+^cells, to further evaluate the role of Ricolinostat in these cells. We confirmed that Ricolinostat promoted the committed differentiation of CD34^+^CD41a^+^ MkPs from CD34^+^CD41a^−^ cells and improved the proliferation capacity of CD34^+^CD41a^+^ MkPs according to CFSE labeling experiment and flow cytometry analysis. It is necessary to expand MkPs as much as possible to produce large numbers of functional MKs. However, the regulatory mechanisms of MkP proliferation and self-renewal are not completely understood. A recent study indicated that MkPs gained expansion capacity with highly enriched expression of genes related to proliferation upon aging [39]. Interestingly, the addition of Ricolinostat to the medium significantly enhanced proliferation-related gene expression in MkPs, further supporting the positive regulatory role of Ricolinostat in increasing the MkP population derived from HSPCs.

We then explored the downstream mechanism of Ricolinostat in enhancing the fate of MkP from CB HSPCs. RNA-seq analysis data indicated that IL-8 and its receptor activity were significantly disturbed by Ricolinostat. It has been reported that megakaryocytic cells derived from CB CD34^+^ cells express CXC chemokines (such as IL-8, neutrophil activating peptide 2, and PF4) [27, 41–45]. Interestingly, our experimental data showed that Ricolinostat significantly inhibited the gene expression of IL-8 and its receptor CXCR2. Ricolinostat, an HDAC6 inhibitor, suppressed the expression of some inflammatory factor genes in human primary chondrocytes [46]. In agreement with the effect of HDAC6 inhibitor in our study, small interfering RNA against HDAC6 in HSPCs had a similar suppression effect on IL-8 and CXCR2 gene expression (Additional file 1: Supplemental Fig. 6). Recent studies indicated that IL-8 and its receptor activation negatively regulate megakaryocytic cell proliferation and differentiation [23, 27, 41–44]. Pharmacological blockade of IL-8 and its receptor interaction by using small molecule inhibitors against CXCR1 and CXCR2 resulted in enhanced production of MK progenitors from umbilical CB CD133^+^ stem cells [27]. Collectively, our data indicate that Ricolinostat might promote the generation of CB CD34^+^ cells into MkPs by inhibiting the interaction of IL-8 and its receptor CXCR2.

To efficiently produce MKs or platelets, many laboratories have employed a stepwise protocol with different cytokine combinations to induce HSPCs to differentiate into MkPs, mature MKs, and platelets. Thus, we further evaluated the production of mature MKs and platelets from these differentiated MkPs using MK maturation medium. The effect of HDAC inhibitors on MK maturation remains controversial [32, 47, 48]. In vivo experiments revealed that the HDAC inhibitors Panobinostat and Romidepsin increased the number of MKs in the mouse bone marrow but reduced the number of proplatelets, suggesting that HDAC inhibitors impair platelet shedding by MKs [32]. In addition, HDAC6 inhibition and genetic knockdown lead to a strong decrease in human proplatelet formation [32]. In contrast, some studies indicated that inhibitors of class II HDACs (e.g., HDAC4, HDAC5, and HDAC6), including LMK235 and tubacin, had little effect on MK maturation. As Ricolinostat may inhibit mature MK production [32], we washed out the small molecule at the end of stage II induction and further observed the differentiation of mature MKs and platelets from these MkPs during stage III. Encouragingly, our data showed that HSPC-derived MkPs induced with Ricolinostat could efficiently differentiated into mature MKs and platelets.

## Conclusions

Our data showed that Ricolinostat promoted MkP differentiation from HSPCs and enhanced the proliferation ability of MkPs. In addition, we developed a differentiation strategy to efficiently induce the generation of MkPs and MKs from HSPCs. Our study will aid in the development of protocols for generating MKs and platelets for clinical use.

## Supplementary Information

**Additional file 1**. Supplemental methods, figures and tables.

## Data Availability

The accession number for the RNA-seq data reported in this study is GEO: GSE182181.

## Abbreviations

MK: Megakaryocyte
HSPC: Hematopoietic stem and progenitor cell
PF4: Platelet factor 4
MkP: Megakaryocyte progenitor cell
CB: Cord blood
HTS: High-throughput screening
SCF: Stem cell factor
TPO: Thrombopoietin
IL: Interleukin
FLT3-L: Flt3-ligand
CFU-MK: Colony-forming unit-MK
qRT-PCR: Quantitative real-time PCR
CFSE: Carboxyfluorescein diacetate succinimidyl ester
CB-PLT: CB-platelet
THBS-1: Thrombospodin-1
IL-8: Interleukin-8
PF4-GFP: PF4 promoter reporter vector with a green fluorescent protein gene
TNC: Total nucleated cell
DEG: Differentially expressed gene
KEGG: Kyoto Encyclopedia of Genes and Genomes
HDAC: Histone deacetylase
GATA1: GATA binding protein 1
RUNX1: RUNX family transcription factor 1
HOXC6: Homeobox C6
PCGF2: Polycomb group ring finger 2
GFI1b: Growth Factor-Independent Protein 1B
GSX2: GS homeobox 2
TAL1: T-cell acute lymphocytic leukemia 1
CXC: C-X-C motif chemokine receptor
FLI1: Fli-1 Proto-Oncogene
BCL2: B-cell lymphoma-2
CDK1: Cyclin-Dependent Kinase 1
CCNB1: Cyclin B1
TERT: Telomerase Reverse Transcriptase
ERG: Ets-related gene
HMGA2: High-Mobility Group AT-hook 2
