# Supplementary material for: Ricolinostat promotes the generation of megakaryocyte progenitors from human hematopoietic stem and progenitor cells
Source: Stem Cell Res Ther. 2022 Feb 5;13:54. doi: 10.1186/s13287-022-02722-5 (PMC8817546; doi:10.1186/s13287-022-02722-5)
Supplement: Supplementary file 1 — Additional file 1. Supplemental methods, figures and tables. [file 13287_2022_2722_MOESM1_ESM.pdf]

Supplemental methods

Morphologic analysis. Cells were rinsed in PBS, cytocentrifuged onto glass slides, stained with Wright–Giemsa solution (Baso, Zhuhai, China), and then observed under light microscopy (Nikon, Tokyo, Japan). NIS-Elements F software (Nikon) was used to capture the images.

Carboxyfluorescein diacetate succinimidyl ester (CFSE) labeling. Carboxyfluorescein diacetate succinimidyl ester (CFSE; Life Technologies, Carlsbad, CA, USA) labeling was conducted using 2 μM CFSE stock according to the manufacturer's protocol for expanded $CD34^+$ cells[1]. CFSE-labeled cells were cultured in differentiation medium with or without Ricolinostat and analyzed using a BD FACSCalibur™.

Immunofluorescence staining. Cultured cells were cytocentrifuged onto glass slides and fixed with 4% paraformaldehyde for 20 min at room temperature (25°C), rinsed with PBS, blocked with 10% donkey serum in PBS for 1 h, and incubated with primary antibodies at 4°C overnight. Labeled isotype-specific secondary antibodies were added when necessary and incubated for 1 h at room temperature (25°C)[2,3]. The cells were counterstained with 4′,6-diamidino-2-phenylindole to visualize the cell nuclei and observed using a confocal microscope (PerkinElmer) and Volocity software (PerkinElmer).

Formation of platelet microaggregates. Washed human CB PLTs and HSPC-PLTs were labeled with PKH67 Green Fluorescent Cell Linker (2 μM, Sigma-Aldrich, St. Louis, MO, USA) and PKH26 Red Fluorescent Cell Linker (2 μM, Sigma-Aldrich), respectively. Fluorescence-labeled human blood platelets were resuspended in modified Tyrode buffer, mixed with fluorescence-labeled HSPC-PLTs, and treated with thrombin (10 U/mL) at 37°C to trigger platelet aggregation. Platelet microaggregates, in 50 μL buffer, were spread onto glass slides and visualized under a confocal fluorescence microscope (PerkinElmer)[4].

Clot formation and retraction. Human CB-platelets (CB-PLTs) or HSPC-PLTs (approximately 1.5×10^7/mL) were resuspended in 200 μL platelet-depleted plasma in siliconized glass tubes. Thrombin (2 U/mL) was added to the cells to induce clot

formation and retraction. The clots were allowed to retract at 37°C for 1 h and then photographed[4].

Transmission electron microscopy. The cell samples were rinsed with PBS and fixed overnight in 3% glutaraldehyde/0.1 M sodium cacodylate, pH 7.4. Following three rinses with sodium cacodylate buffer, the samples were postfixed for 1 h in 1% osmium tetroxide/0.1 sodium cacodylate buffer. After rinsing in deionized water, the samples were dehydrated and embedded in Polybed 812 epoxy resin (Polysciences, Inc., Warrington, PA, USA). The samples were sectioned perpendicularly to the substrate at 70 nm using a diamond knife. Ultrathin sections were collected on 200 mesh copper grids and stained with 4% aqueous uranyl acetate for 15 min, followed by Reynolds' lead citrate for 7 min. Samples and stained sections were observed using an H7650 transmission electron microscope (HITACHI, Tokyo, Japan) operating at 80 kV (H7650 Electron Microscopy) and photographed using an AMT XR16M CCD digital camera and AMT Capture Engine Software Version 600.259 (Advanced Microscopy Techniques, Woburn, USA)[1].

Ploidy analysis. Cells were immunostained for CD41a and CD42b expression. After the cells were fixed with 4% paraformaldehyde, they were incubated with 50 μg/mL propidium iodide (Beyotime, Beijing, China) for 30 min before flow cytometry analysis.

Measurement of thrombospodin-1, PF4, and IL-8. We performed enzyme-linked immunosorbent assay (ELISA) to quantify thrombospodin-1 (THBS-1), PF4, and IL-8 in the culture suspension and cell lysates of 1×10^6 cells from each group. A Human THBS-1 ELISA Kit (Solarbio, Beijing, China), PF4 ELISA Kit (Boster, Wuhan, China), and IL-8 ELISA Kit (Solarbio, Beijing, China) were used according to the manufacturer's protocols[5].

RNA-sequencing of $CD34^+CD41^+$ cells. $CD34^+CD41^+$ cells were sorted by flow cytometry on day 7. After 7 days of Ricolinostat treatment, the cells were collected and centrifuged at 2000 rpm. For RNA-sequencing (RNA-seq) samples, total RNA was isolated using an RNeasy extraction kit and treated with DNase[1]. RNA-seq was performed using a Novaseq 6000 S4 platform (Illumina, San Diego, CA, USA). Low-quality raw reads were filtered. Clean data were mapped to the reference genome using

HISAT2 v2.1.0. The read counts of each gene were summarized using HTSeq (http://www-huber.embl.de/users/anders/HTSeq/doc/overview.html). DEGseq2 v1.6.3 was used to detect differentially expressed genes (fold-change above 1 with adjusted p-values below 0.01). RNA-seq data were analyzed using Annoroad Gene Tech Co., Ltd. (Beijing, China).

Small interfering RNA transfection. Cells were seeded into 6-well plates and transfected with 100 pmol of small interfering RNA (GenePharma, Shanghai, China) using Lipofectamine®2000 Reagent (Invitrogen, Waltham, USA) according to the manufacturer's protocol**.**

References:

1. Wang Y, Qin J, Wang S, et al. Conversion of Human Gastric Epithelial Cells to Multipotent Endodermal Progenitors using Defined Small Molecules. Cell Stem Cell. 2016;19:449-461.
2. Fathi E, Farahzadi R, Vietor I, Javanmardi S. Cardiac differentiation of bone-marrow-resident c-kit(+) stem cells by L-carnitine increases through secretion of VEGF, IL6, IGF-1, and TGF- beta as clinical agents in cardiac regeneration. J Biosci. 2020;45.
3. Fathi E, Vietor I. Mesenchymal Stem Cells Promote Caspase Expression in Molt-4 Leukemia Cells Via GSK-3alpha/Beta and ERK1/2 Signaling Pathways as a Therapeutic Strategy. Curr Gene Ther. 2021;21:81-88.
4. Zhang B, Wu X, Zi G, et al. Large-scale generation of megakaryocytes from human embryonic stem cells using transgene-free and stepwise defined suspension culture conditions. Cell Prolif. 2021;54:e13002.
5. Fathi E, Valipour B, Sanaat Z, Nozad CH, Farahzadi R. Interleukin-6, -8, and TGF-beta Secreted from Mesenchymal Stem Cells Show Functional Role in Reduction of Telomerase Activity of Leukemia Cell Via Wnt5a/beta-Catenin and P53 Pathways. Adv Pharm Bull. 2020;10:307-314.

Supplemental Figure 1

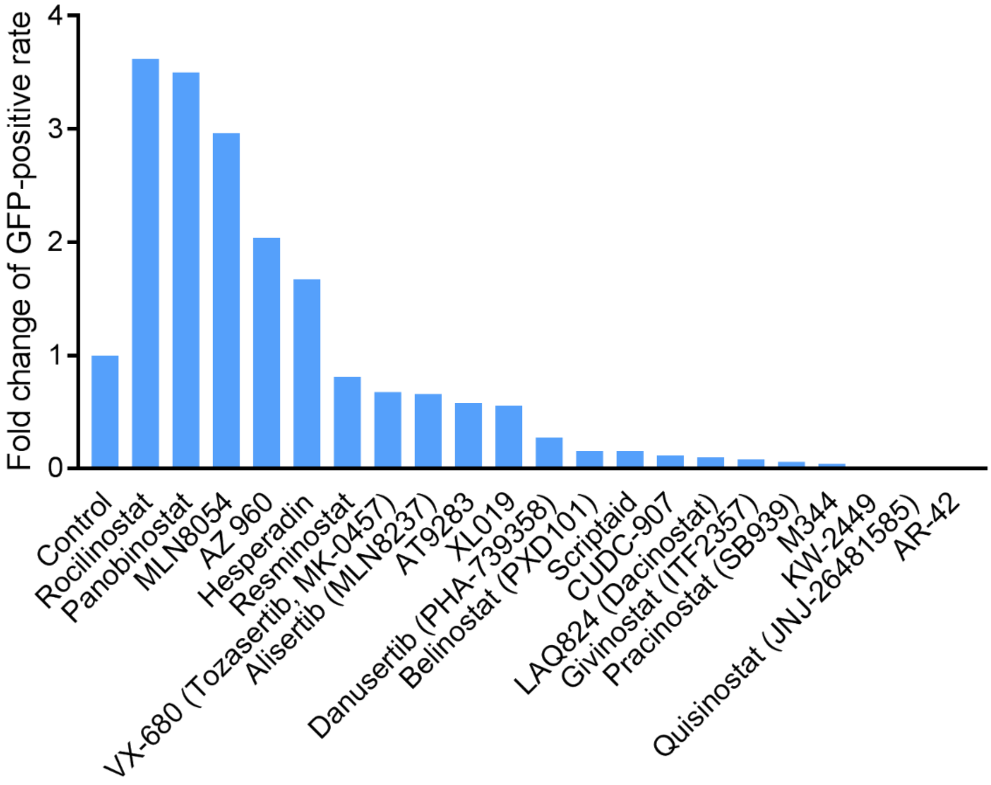

Supplemental Figure 1. Flow cytometry analysis of GFP expression in K562 cells treated with candidate small molecules.

Supplemental Figure 2

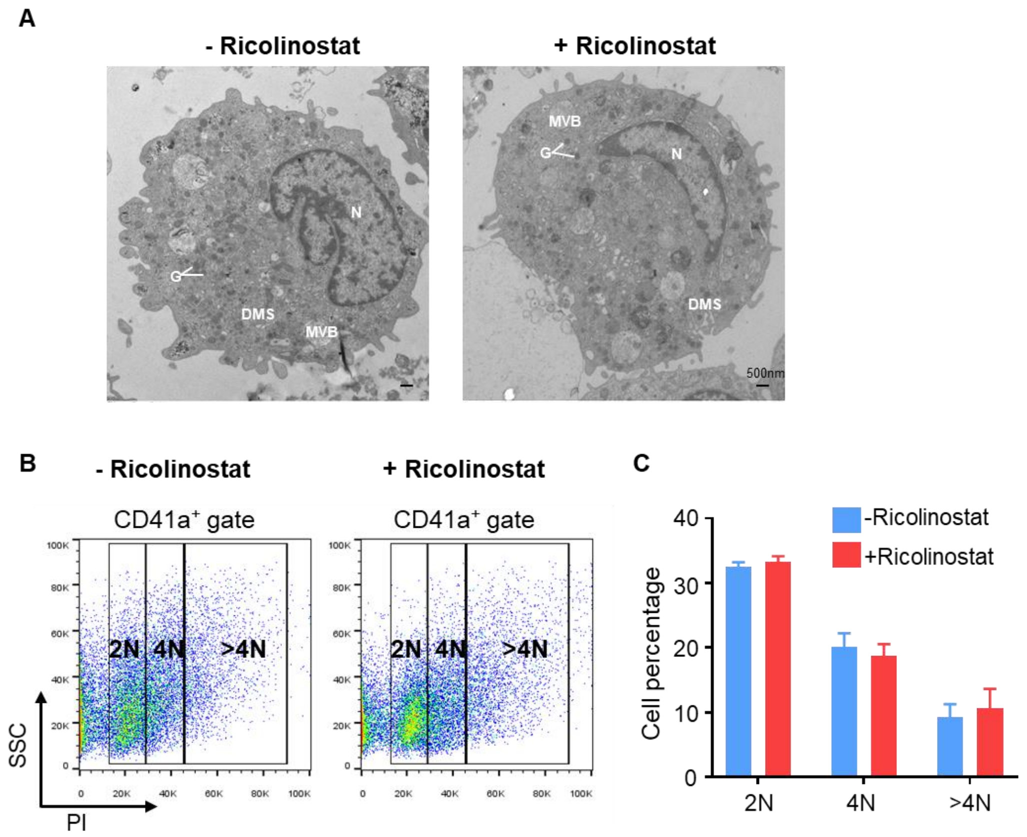

Supplemental Figure 2.

(A) Transmission electron micrographs of cells on day 21. N, nucleus; DMS, demarcation membrane system; G, granules; MVB, multivesicular body. Scale bars, 500 nm.

(B - C) DNA ploidy analysis of the differentiated MKs on day 21. (B) Flow cytometry plot of 2N, 4N, and >4N cells among CD41a+ cells. (C) The percentages of 2N, 4N, and >4N cells among CD41a+ cells.

Supplemental Figure 3

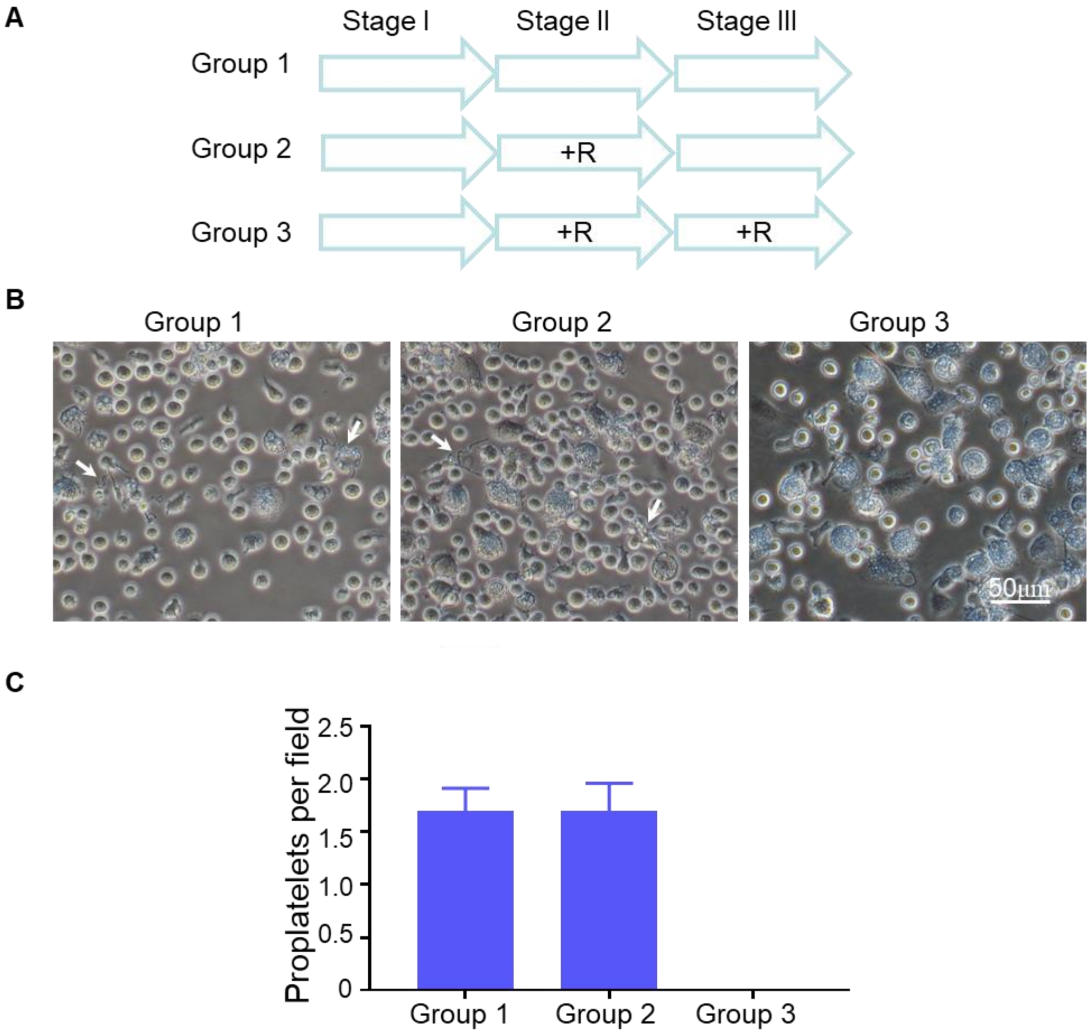

Supplemental Figure 3. Proplatelet formation analysis.

(A) Schematic diagram showing Ricolinostat treatment at different stages in each group. R, Ricolinostat.

(B) Representative phase contrast picture of spontaneous proplatelet-forming MKs.

(C) Proplatelet number per field in each group.

Supplemental Figure 4

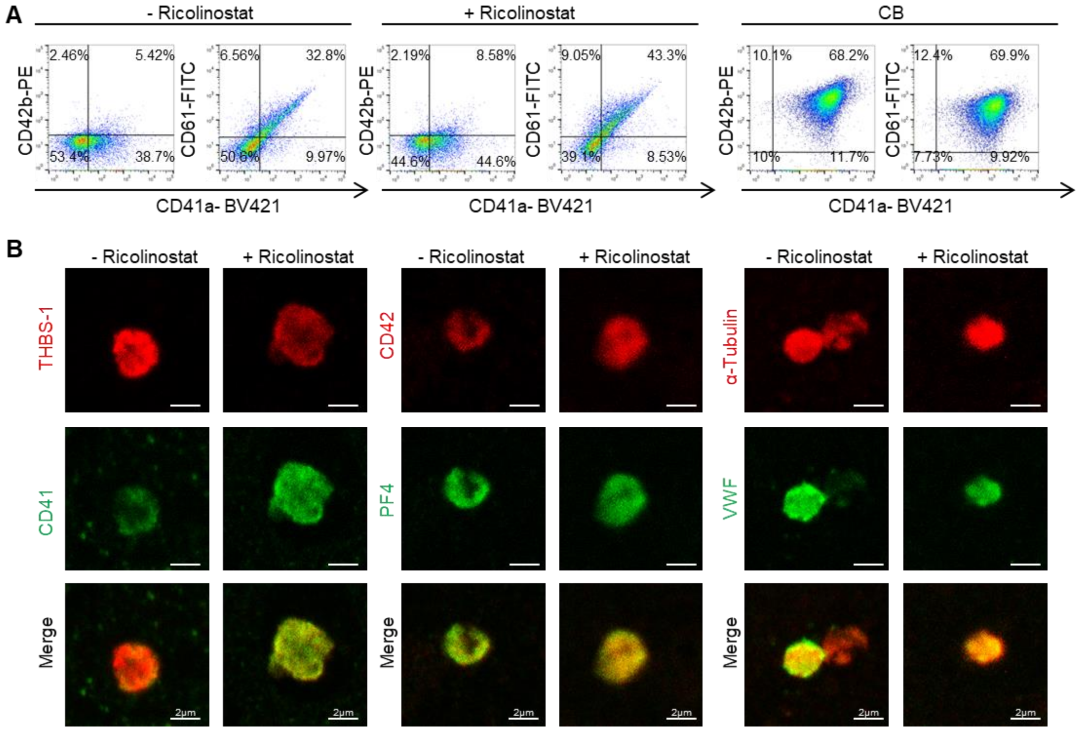

Supplemental Figure 4. Platelet production.

(A) Analysis of platelet production in different treatment groups by flow cytometry.

(B) Immunofluorescence staining analysis showed the expression and location of CD41, CD42b, α-tubulin, VWF, PF4, and THBS-1 in platelets produced by different treatment groups. Scale bars, 2 μm.

Supplemental Figure 5

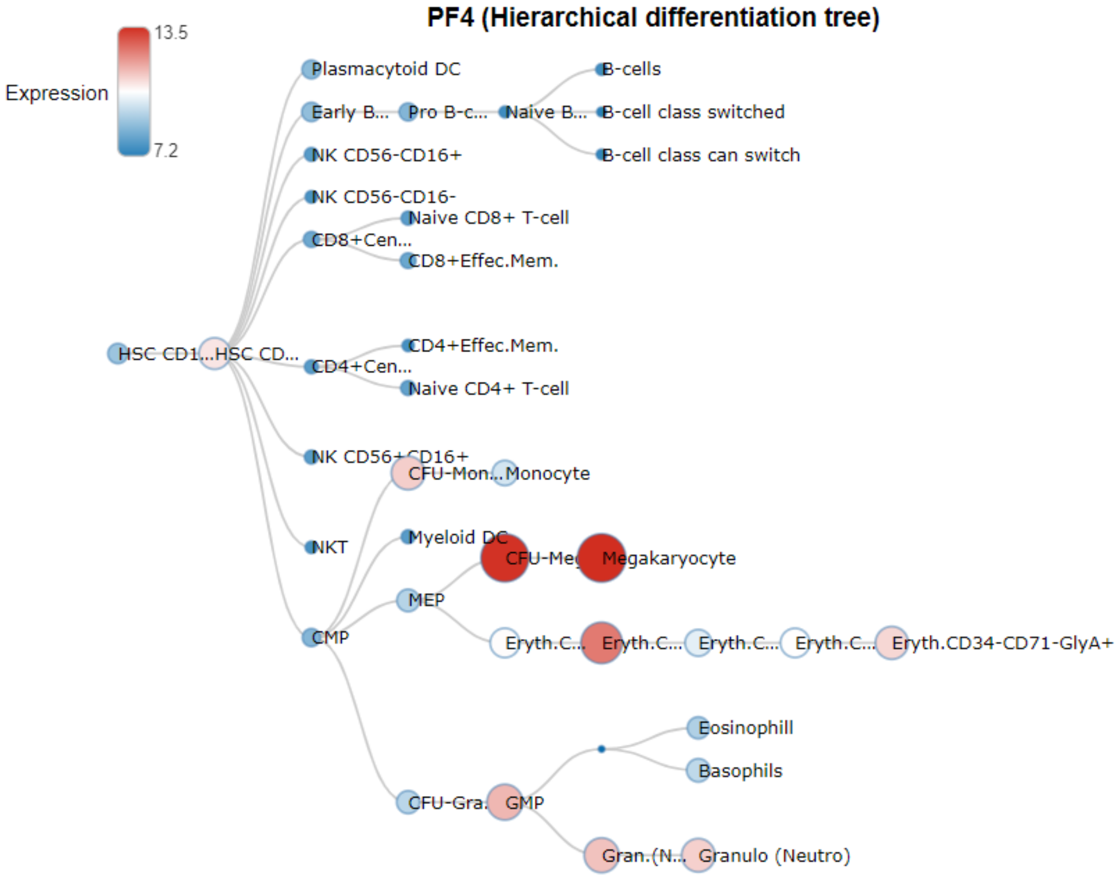

Supplemental Figure 5. The PF4 mRNA expression levels in the hematopoietic lineages according to DMAP dataset in Bloodspot database (http://servers.binf.ku.dk/bloodspot).

Supplemental Figure 6

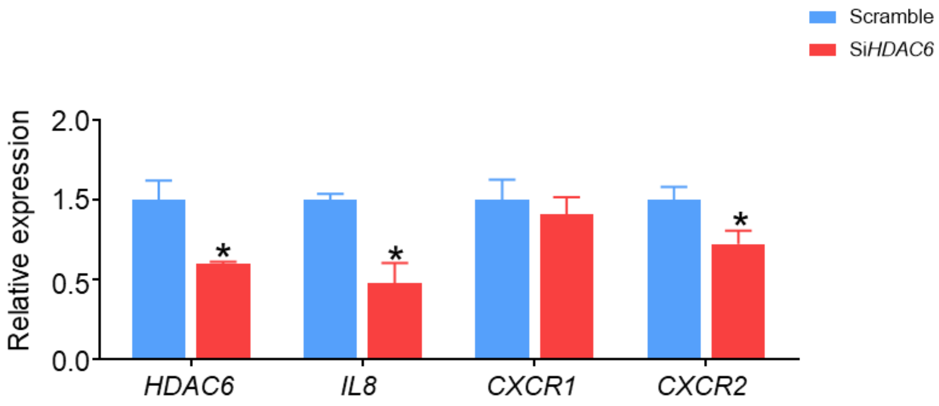

Supplemental Figure 6. Knockdown of HDAC6 decreased the expression of IL8 and CXCR2 genes. Expanded CD34$^+$ cells on day 7 were transiently transfected with siHDAC6 or scramble siRNA. The qRT-PCR analysis of the indicated genes were performed at 72 h after transfection.

Table S1. Antibodies used in this study.

Primary Antibody

Antibody	Company	Product Code	Ig Species	Dilution
vWF	Chemicon	AB7356	Rabbit	100(ICC)
α-Tubulin	Sigma	T9026	Mouse	500(ICC)
Thrombospondin	Abcam	ab1823	Mouse	50(ICC)
PF4	Boster	BA4122	Rabbit	200(ICC)
CD41	Abcam	ab134131	Rabbit	100(ICC)
CD42b	eBioscience	14-0429-82	Mouse	100(ICC)

Conjugated antibody

Antibody	Company	Product Code	Ig Species
CD38-PE	BD	555460	Mouse
CD34-PE-cy7	BD	560710	Mouse
CD41a-APC	eBioscience	17-0419-42	Mouse
CD42b-PE	eBioscience	12-0429-42	Mouse
CD45RA-PF-CF594	BD	562298	Mouse
CD61-FITC	eBioscience	11-0619-42	Mouse
CD123-BV421	BD	562517	Mouse
CXCR1-FITC	eBioscience	11181942	Mouse
CXCR2-PE	eBioscience	12182942	Mouse

Secondary Antibody

Secondary Antibody	Company	Product Code	Dilution
Alexa Fluor® 568 Donkey Anti-Mouse IgG (H+L)	Invitrogen	A10037	400
Alexa Fluor® 488 Donkey Anti-Rabbit IgG (H+L)	Invitrogen	A21206	400

Table S2. Primers used in this study.

Gene	Forward	Reward
GAPDH	GAGTCAACGGATTTGGTCGT	TTGATTTTGGAGGGATCTCG
CMPL	TAAACCAGACTCGGACTCAGC	TAAACCAGACTCGGACTCAGC
FOG1	CGTGCTTCGAGTGCGAGAT	GGCCTGAACAGTAGAGGCG
MAFK	GAGAGTGGCTCACACGTCG	CTGCTCACCGTCAAATGATGG
RUNX1	CTGCCCATCGCTTTCAAGGT	GCCGAGTAGTTTTCATCATTGCC
TAL1	AGCCGGATGCCTTCCCTAT	GGGACCATCAGTAATCTCCATCT
HOXC6	ACAGACCTCAATCGCTCAGGA	AGGGGTAAATCTGGATACTGGC
PCGF2	CGGACTACACGGATCAAAATCA	GGCGTCGATGAAGTACCCC
GFI1b	GCAGGAAGATGAACCGCTCT	CCAGGCACTGGTTTGGGAA
IL8	CACCGGAAGGAACCATTCTC	AATCAGGAAGGCTGCCAAGA
CXCR1	CTGACCCAGAAGCGTCACTTG	CCAGGACCTCATAGCAAACTG
CXCR2	CCTGTCTTACTTTTCCGAAGGAC	TTGCTGTATTGTTGCCCATGT
CDC25A	ACAAACCTTGACAACCGATGC	ACTGACCGAGTGCTGGAGCT
CDK1	GGATGTGCTTATGCAGGATTCC	CCATGTACTGACCAGGAGGGATAG
BCL-2	GAGGATTGTGGCCTTCTTTG	ATCACCAAGTGCACCTACCC
PCNA	TGTCGATAAAGAGGAGGAAGCTG	TGAAGAGAGTGGAGTGGCTTTTG
CCNB1	TTGGGGACATTGGTAACAAAGTC	ATAGGCTCAGGCGAAAGTTTTT
TERT	TATGGCTGCGTGGTGAACTTG	CATAGCTGGAGTAGTCGCTCT
PF4	AGCCTGGAGGTGATCAAGG	CCATTCTTCAGCGTGGCTA
